# Internal Medication in Gonorrhea

**Published:** 1908-02

**Authors:** 


### Internal Medication in Gonorrhea

Joseph Piket, assistant at the Allgemeine Poliklinik, Vienna, (*Berichte d. Versammlung Deut. Naturforscher u. Aerzte,* September, 1907,) says the treatment of gonorrhea taxes all the therapeutic resources at our command, among which internal remedies by no means rank as the least important. Of the latter, the balsams until lately held the dominant position. But in the large doses in which they have to be given. they disturb the gastrointestinal tract and, by the excretion of resinous acids. irritate the kidneys, while normal renal function is indispensable in the internal treatment of gonorrhea. Occasionally, too, a balsamic erythema or urticaria is observed. As, moreover, the balsams are powerless to prevent the development of gonorrhea, it was most desirable to find a more effective adjuvant to or, under proper circumstances, a substitute for the injection treatment.

These efforts have found successful culmination in arhovin, an addition product of diphenylamine and esterified thymyl-benzoic-acid. By reason of its strongly antiseptic components, arhovin possesses a marked disinfectant action, which after its exhibition is still greater, as the remedy,—that is, its decomposition products,—renders the urine acid, clears and imparts bacterio-inhibitory properties to that excretion, and thus combats the pus cocci. Arhovin is not toxic, has no harmful effect on stomach or intestines, and does not irritate the kidneys. According to Burchard-Schlockow's work, it is absorbed in 20 to 25 minutes, and is excreted in altered form. The acidity of arhovin urine is so marked that usually it remains acid for 14 to 18 days. Piket's experience with it extends over two years and includes a large number of severe cases, both of acute and chronic gonorrhea, gonorrheal cystitis and complications in the female. He summarises his opinion as follows: "Arhovin is readily taken and well borne, has no deleterious effect of any kind, limits secretion, hinders gonococcal growth, and possesses a marked sedative action. It is a valuable addition to the armamentarium,—a remedy that no physician will want to dispense with, for a test leads to its permanent adoption."

---

INFLUENZA OF THE NOSE, THROAT, AND LARYNX.—W. Sohier Bryant of New York describes the course of influenza and the complications involving the nose, throat and larynx. Pressure in the nose causes reflex symptoms in the higher centers of the cortex and brain. The ostia of the various sinuses are closed and catarrhal inflammation of those cavities may ensue. The frontal and maxillary sinuses cause frontal and facial pain and tenderness. When the ethmoidal and sphenoidal sinuses are involved there is extreme intranasal tenderness and discharge, with pain deep in the head. In the pharynx tonsillar inflammation and abscess may occur. The larynx when involved, shows its inflammation by irritating cough and pain. Prognosis for recovery is good. Treatment is abortive, local and general. Abortive treatment consists of the application of astringents, preferably silver nitrate to the mucous membranes. Irrigation with saline solutions and peroxide of hydrogen are useful when there is pus. Sinus complications may be helped by hot irrigations of the nasal cavities.—*Medical Record*, January 11, 1907.

THE RENAL COMPLICATIONS AND SEQUELAE OF INFLUENZA.—Heinrich Stern of New York believes that renal complications after influenza have not been given the importance that is desirable. Slight complications in the form of renal congestion are frequent. Acute nephritis is not common, but the bacilli may be retained for years in the body and become periodically active, and affect the kidneys. Aggravation of a previous existing renal disease is frequent and of importance as a matter of prognosis. Post-influenzal nephritis occurs after the acute symptoms have subsided. This form is chronic and a phenomenon of reinfection. It is really a sequela and is of the character of chronic interstitial degeneration.—*Medical Record*, January 11, 1907.

